# Soil Fungal Communities Across Contrasting Land-Use Systems in an Intensively Managed Cerrado Landscape

**DOI:** 10.3390/jof12050346

**Published:** 2026-05-07

**Authors:** Jefferson Brendon Almeida dos Reis, Thayssa Monize Rosa de Oliveira, Samia Gomes-da-Silva, Maria Regina Silveira Sartori da Silva, Fabyano Alvares Cardoso Lopes, Alessandra Monteiro de Paula, Nadson de Carvalho Pontes, Helson Mario Martins do Vale

**Affiliations:** 1 University of Brasilia, Institute of Biological Sciences, Brasília 70910-900, DF, Brazil; jeffersonalmeidareis@gmail.com (J.B.A.d.R.);; 2 Centro de Excelência em Bioinsumos (CEBIO), Instituto Federal Goiano, Campus Morrinhos, Morrinhos 75650-000, GO, Brazil; 3 University of Brasilia, Faculty of Agronomy and Veterinary Medicine, Brasília 70910-900, DF, Brazilalessandramp@unb.br (A.M.d.P.); 4 Laboratory of Microbiology, Federal University of Tocantins, Porto Nacional 77500-000, TO, Brazil

**Keywords:** cover crop, rotation, core-microbiota, *Fusarium*, savannah, *Trichoderma*

## Abstract

Understanding how agricultural soil management affects soil fungal communities is essential for assessing the resilience of biodiversity hotspots such as the Brazilian Cerrado. In this study, we characterized fungal community structure across three contrasting land-use systems within the same agricultural landscape: a native Cerrado remnant, a cover-cropping system, and a spatially isolated potato monoculture field. The soil’s chemical and enzymatic characteristics differed from one another and were clustered by area. However, the same pattern was not observed for the fungal community. Alpha-diversity indices did not differ significantly among sites, although native Cerrado soils showed slightly higher richness and evenness. Beta-diversity analyses based on Bray–Curtis and Jaccard distances, supported by NMDS, ANOSIM, beta-dispersion, and PERMANOVA, indicated no significant compositional differences among communities. Core-mycobiota analysis identified 157 shared ASVs, including genera such as *Fusarium*, *Cladosporium*, *Chrysosporium*, *Trichoderma*, and *Clonostachys*. As a preliminary assessment based on a limited spatial design and sequencing-based inference, these findings should be interpreted with caution. These results underscore the need for further research on the mechanisms driving fungal dispersal, edge effects, and the long-term impacts of agricultural land-use on fungal diversity and ecological integrity in the Cerrado.

## 1. Introduction

Fungi constitute one of the most abundant and functionally diverse groups of eukaryotes in soil [1,2], participating in some biogeochemical cycles [3], such as the decomposition of organic matter [4], and in interactions with other organisms [5,6]. Fungal diversity is related to the stability and resilience of soil ecosystems [7] and can influence the quality of the soil environment and the sustainability of production systems [8,9]. Additionally, numerous studies demonstrate that the taxonomic, genetic, trophic, and functional composition of soil fungal communities varies in response to soil physicochemical properties, vegetation type, topography, and management history [10,11,12]. Thus, understanding how the composition and diversity of fungal communities vary across different soil types and under different management conditions is essential to assess the impacts of agricultural practices on soil health and ecological balance [13,14]. In recent decades, there has been growing scientific interest in biotic homogenization processes in microbial communities, especially in environments subjected to agricultural intensification or urbanization [15,16,17,18]. Biotic homogenization does not necessarily imply an absolute loss of diversity but rather a reduction in dissimilarity between originally distinct communities, which become more similar to each other [19,20]. Recent evidence shows that agricultural practices can promote this process by altering environmental filters and by facilitating the flow and predominance of certain microbial taxa [16,18].

Potato (*Solanum tuberosum*) cultivation, characterized by high nutritional demands [21] and susceptibility to various fungal and oomycete pathogens [22], constitutes an emblematic example of an intensified agricultural system [23,24,25]. Diseases caused by *Fusarium* [26], *Rhizoctonia* [27], and *Phytophthora infestans* [28] require constant management, with frequent application of chemical and biological inputs in conventional farming systems [22]. In contrast, areas managed with cover crops tend to exhibit greater accumulation of organic matter [29,30,31], increased microbial biomass [32], and to favor beneficial fungal taxa [33], such as *Trichoderma* [34] and *Mortierella* [33]. These systems represent extremes of a management gradient, offering a valuable opportunity to investigate how agricultural practices modulate the structure of fungal communities.

In the context of the Brazilian neotropical savannah (Cerrado), recognized as a global biodiversity hotspot [35], this gradient becomes even more relevant. Despite its high microbial richness in both soil [36] and plant life [37], the Cerrado has been extensively converted into agricultural areas and pastures, resulting in profound alterations in the chemical and biological properties of the soil [38,39,40]. Thus, even fragments that maintain native vegetation are frequently located near intensive systems, becoming subject to edge effects [41,42] and gradual modifications in their microbiota [38,43,44,45]. Such processes can compromise the ecological uniqueness of the Cerrado by bringing the microbial structure of these areas closer to that observed in cultivated soils.

Studies investigating the impact of agriculture on soil microbial communities in the Cerrado biome have demonstrated differences in the taxonomic composition of communities in different environments (native areas or agricultural systems), as well as variation in community composition between environments [38,43,44,45]. Souza and Propópio [44] found evident differences in community structure through alpha and beta-diversity analysis when comparing soil microbial communities present in areas of native Cerrado vegetation, pasture, and soybean cultivation. Furthermore, edge effects can influence the composition and diversity of fungal communities [46,47]. However, more recent evidence suggests that agricultural intensification can reduce these differences, promoting the homogenization of microbial communities [15,16,17,18]. The proximity of native Cerrado remnants to highly intensive agricultural areas, such as potato fields and post-harvest cover cropping systems, may facilitate the flow of fungal propagules between environments and reduce community dissimilarity, providing an opportunity to investigate the extent of similarity among these communities.

Based on this context, we hypothesize that soil fungal communities in a native Cerrado remnant will exhibit high taxonomic overlap with both adjacent and spatially distant agricultural systems, resulting in reduced compositional differentiation among communities. Therefore, our study aims to characterize and compare soil fungal communities across three contrasting environments: a native Cerrado remnant within an intensively cultivated landscape, a nearby cover cropping system, and a spatially isolated potato field. Within this framework, we assess patterns of taxonomic overlap among communities to evaluate the potential influence of agricultural practices on fungal community structure. As an exploratory (pilot) study, our work provides a baseline for future research investigating the effects of biotic homogenization, edge effects, and propagule flow on soil fungal community structure in Cerrado ecosystems.

## 2. Materials and Methods

### 2.1. General Description of the Areas and Soil Sampling

**Figure 1:**
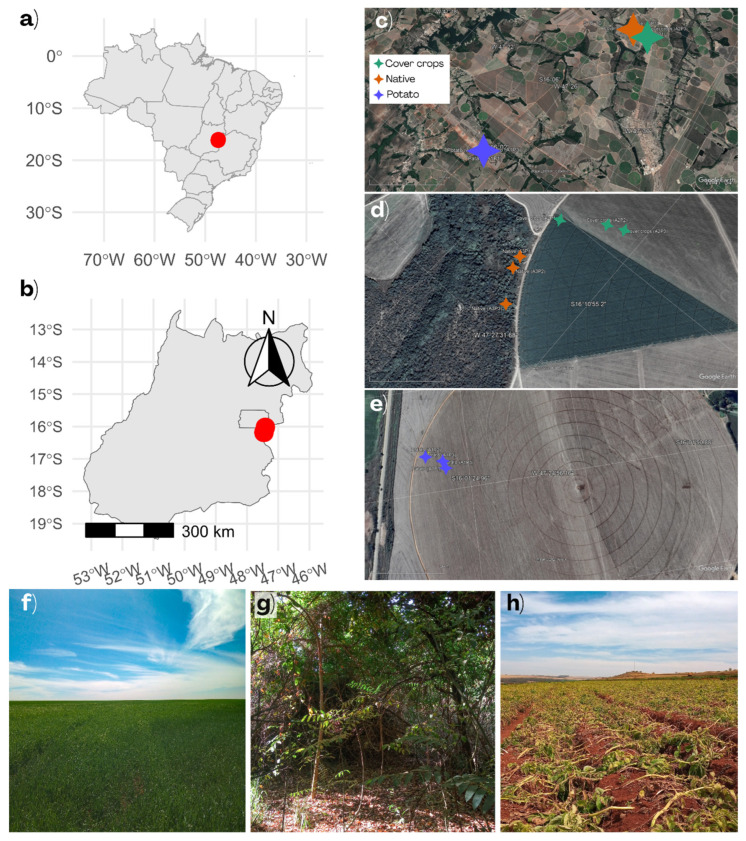
Location of the study area and land-use systems in the Brazilian Cerrado. (**a**) Map of Brazil highlighting the study area. (**b**) Location of the sampling site (red point) within the state of Goiás. (**c**–**e**) Satellite images “Google Earth: https://earth.google.com/web/ (accessed on 31 August 2022)” showing the geographic coordinates of the sampling points for each land-use system: Cover Crops, Native, and Potato. (**f**–**h**) Representative photographs of each land-use system: (**f**) Cover Crops area; (**g**) Native area; (**h**) Potato area.

To investigate the soil fungal community, three areas were selected within the same agricultural property in Cristalina, Goiás, Brazil (Figure 1a,b). This municipality is a region dominated by intensive mechanized agriculture and cattle pastures [48,49]. The climate is Aw (Köppen), with a tropical savanna pattern: wet season from October to April and dry season from May to September. Sampling occurred in August 2022, during the dry season. In the month the samples were collected (August 2022), no precipitation was recorded at the conventional weather station in the Federal District, representing a 100% deficit compared to the climatological normal (1991–2020), estimated at 16.3 mm. Up to 31 August 2022, 116 consecutive days without rain were recorded, with the last event observed on May 7, with an accumulated rainfall of 13.3 mm. The average monthly temperature was 21.3 °C, slightly above the climatological normal (21.0 °C). The average minimum temperature was 14.9 °C, lower than the historical average (15.3 °C), while the average maximum temperature reached 28.2 °C, exceeding the reference value (27.4 °C) (INMET, Brazil). The areas included a commercial potato field (PC) near harvest, a 45-day-old cover crop mixture of rye (*Secale cereale*) and buckwheat (*Fagopyrum esculentum*) (CC), and a native Cerrado remnant (NC, Cerrado *sensu stricto*) within the monoculture rotation system (Figure 1f–h) [50]. Cultivated areas were irrigated via a central pivot. PC was located ~18.5 km from CC and NC, representing a spatially isolated system, whereas CC and NC were <60 m apart (Figure 1c–e).

**Figure 2:**
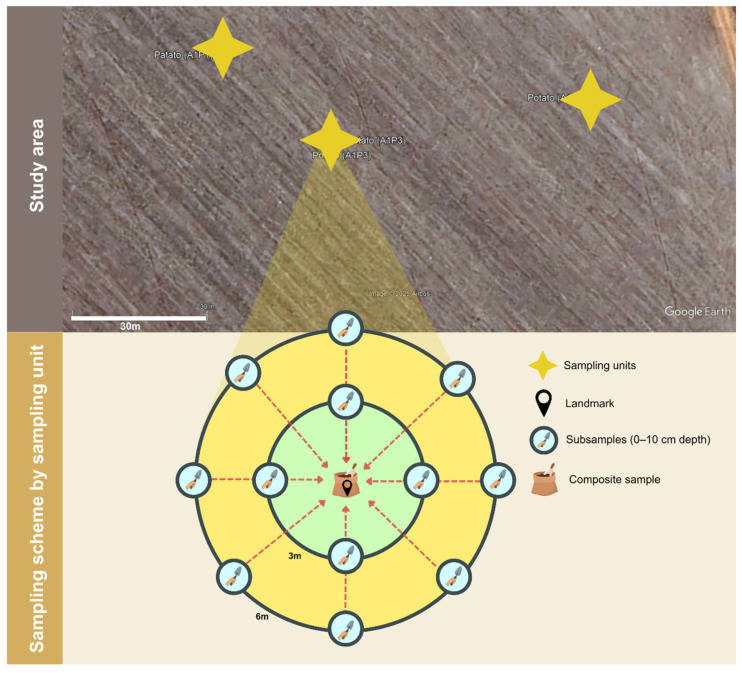
Soil sampling scheme.

Three sampling points were established per area, with soils collected approximately 30 m from the edges, defined here as the boundaries between each sampling area and adjacent land-use systems. Sampling points were also spaced at a minimum distance of ~30 m apart (range ~35–150 m). While this distance was intended to reduce edge effects, we acknowledge that some influence from edges cannot be completely ruled out, particularly in fungal communities [46,47]. At each point, twelve subsamples were collected in two concentric circles (radii 3 and 6 m) around a georeferenced central location and combined into a composite sample (0–10 cm depth), yielding three composite samples per area (Figure 2) [51]. Samples were stored at 4 °C until analysis. In the three study areas, the soil was classified Red-Yellow Latosol (Latossolo Vermelho-Amarelo) [52], corresponding to Ustox (USDA system) [53].

### 2.2. Soil Chemical and Enzymatic Analyses

Detailed soil analyses can be found in dos Reis et al. [50]. Chemical characterization was performed in triplicate, including pH, exchangeable Al, Ca, Mg, extractable P and K, total C, Zn, titratable acidity (H + Al), and effective cation exchange capacity (CEC), following standard methods [54]. Soil moisture was determined after oven-drying at 105 °C. Enzymatic activities of β-glucosidase, arylsulfatase, and acid phosphatase (related to C, S, and P cycles, respectively) were measured in triplicate at their optimal pH values (6.0, 6.5, and 5.8, respectively) including controls, following Tabatabai [55].

### 2.3. DNA Extraction from Soil Samples, Sequencing, and Bioinformatic Processing

Extraction of DNA from soil samples was performed with the FastDNA SPIN Kit for Soil (MP Biomedicals, Santa Ana, CA, USA) in accordance with the supplier’s instructions to ensure consistent recovery of microbial DNA. DNA quality and quantity were determined with a NanoDrop spectrophotometer (Thermo Scientific, Wilmington, DE, USA), and integrity was verified by 1% (*w*/*v*) agarose gel electrophoresis. The ITS2 region was amplified using the primer pair ITS3-2024F (5′-GCATCGATGAAGAACGCAGC-3′) and ITS4-2409R (5′-TCCTCCGCTTATTGATATGC-3′). Sequencing was performed on an Illumina MiSeq platform (Illumina, San Diego, CA, USA) at Novogene (Beijing, China). Raw reads were subjected to quality control and adapter trimming using BBDuk v38.34 (BBMap; Bushnell B., sourceforge.net/projects/bbmap) with the following parameters: removal of Illumina adapters and PhiX Control v3 sequences; ktrim = l; k = 23; mink = 11; hdist = 1; minlen = 50; --tpe; --tbo; qtrim = rl; trimq = 20; ftm = 5; maq = 20. Cleaned paired-end reads were then imported into QIIME2 v2022.11 (https://qiime2.org) for bioinformatic processing [56]. Sequence denoising, dereplication, merging of paired-end reads, and chimera removal were conducted using the DADA2 plugin [57]. Taxonomic assignment of amplicon sequence variants (ASVs) was performed with the qiime2-feature-classifier plugin [58] using the Naive Bayes classifier trained on the UNITE database for all eukaryotes (ITS, version 9.0) [59].

### 2.4. Data Analysis

Fungal communities analyses and analyses of soil chemical and enzymatic characteristics were performed in RStudio (v.4.4.0, 2024-04-24 ucrt) [60]. To assess whether the similarity of fungal communities across areas with different soil characteristics reflects biotic homogenization processes, we used chemical and enzymatic soil data from the same areas [50]. We performed multivariate analyses to determine whether the areas form distinct clusters. The numerical data were organized into a matrix and standardized using the “scale()” function, ensuring that all variables contributed equally to the subsequent analyses. We conducted a Principal Component Analysis (PCA) with the “prcomp()” function using internal standardization, and visualized the results with “fviz_pca_ind()” from the factoextra package (v.1.0.7), representing the samples in a two-dimensional space with the aid of ggplot2 (v.3.5.2). To summarize the multivariate profile of each area, we constructed radar charts from the min–max–normalized variables, aggregated by area using functions from the dplyr (v.1.1.4) and tidyr (v.1.3.1) packages, and plotted them with “radarchart()” from the fmsb package (v.0.7.6). We also examined the similarity structure among samples using hierarchical cluster analysis: the Euclidean distance matrix was computed with “dist()”, followed by the complete-linkage method with “hclust()”, and the dendrogram was visualized using ggdendro (v.0.2.0) integrated with ggplot2.

Tables of ASVs with their respective taxonomic assignments, together with environmental metadata, were integrated to generate a “phyloseq” object using the “phyloseq” package (v.1.52.0). The “phyloseq” object was first filtered to remove ASVs not assigned to the kingdom Fungi. It was then converted to the microeco format using the file2meco package (v.0.80). To evaluate whether differences in sequencing depth could bias downstream analyses and whether rarefaction should be applied [61,62,63], we compared the number of non-chimeric reads obtained per sample after denoising. The depths were relatively homogeneous across samples, ranging from approximately 83,000 to 161,000 reads, with a maximal difference of ~1.94-fold between the shallowest and deepest libraries (Supplementary Material—Supplementary Table S1). This variation falls well below the threshold typically associated with biases in diversity estimates, approximately an order of magnitude (≈10×) [64]. Therefore, rarefaction was not applied [64,65]. To further validate sequencing coverage, rarefaction curves were generated for each sample using the vegan (v.2.6-8) and iNEXT (v.3.0.1) packages, confirming that all libraries reached a plateau, indicating sufficient sampling depth (Supplementary Material—Supplementary Figure S1a,b). Subsequently, a minimum relative abundance threshold of 0.00001 was applied to exclude low-representation ASVs. This filtering step reduced the dataset from 704 to 598 ASVs, removing potential noise while retaining the majority of the diversity for downstream analyses.

Relative abundance at different taxonomic levels was calculated using the trans_abund class of the microeco package (v.1.9.1). Differential abundance was calculated using the LEfSe method implemented in the “trans_diff” class of the microeco package. We used the “trans_func” class from the microeco package together with the FungalTraits database to assign primary lifestyle categories. Differences in primary lifestyle categories among areas were tested using the Kruskal–Wallis test, followed by post hoc comparisons with *p*-value correction. Pearson correlation analyses were used to examine the relationships between fungal genera relative abundance and primary lifestyle with soil chemical and enzymatic variables.

Alpha and beta diversity analyses were performed using the “trans_alpha” and “trans_beta” classes, respectively. These analyses were conducted at both the ASV and genus levels. Alpha diversity was assessed using observed richness, Shannon diversity, Simpson diversity, and Pielou’s evenness indices. Beta diversity was evaluated based on Bray–Curtis and Jaccard distance matrices to assess community similarity and dissimilarity among the three experimental areas, and visualized through Non-metric Multidimensional Scaling (NMDS). When NMDS exhibited stress values above the acceptable threshold, Principal Coordinates Analysis (PCoA) was used to represent the community structure. Intra- and inter-group dispersion and similarity were tested using β-dispersion (“beta_disper”; 999 permutations) and ANOSIM (Analysis of Similarities; 999 permutations). Finally, PERMANOVA (Permutational Multivariate Analysis of Variance; 999 permutations) was applied to assess statistically significant differences in fungal community composition among the evaluated areas.

To identify stable and consistent taxa across all samples, allowing us to distinguish resident members of the mycobiota from sporadic or transient taxa, we performed a core mycobiota analysis across the three areas. From an ecological perspective, especially in changing environments, this analysis can provide insights into the most stable taxa and their potential functional roles. The core mycobiota of fungal communities among the areas was identified using ASV data processed in phyloseq. To address the presence of zero values, which are common in compositional mycobiota datasets and can interfere with downstream transformations, a pseudo-count (1 × 10^−6^) was added prior to conversion into relative abundances. This prevents computational issues and enables subsequent analyses using the “microbiome::transform function” (v.1.26.0). The taxonomic table was cleaned and standardized by correcting column names and removing unnecessary prefixes (k__, p__, etc.). Subsequently, the function “microbiome::add_besthit” was used to associate each ASV with its most probable taxonomic assignment. The core mycobiota was defined as the set of ASVs present in at least 50% of samples with a minimum relative abundance of 0.01%. The composition of the core mycobiota was analyzed and visualized using line plots and heatmaps.

## 3. Results

### 3.1. Chemical and Soil Enzyme Analysis

**Figure 3:**
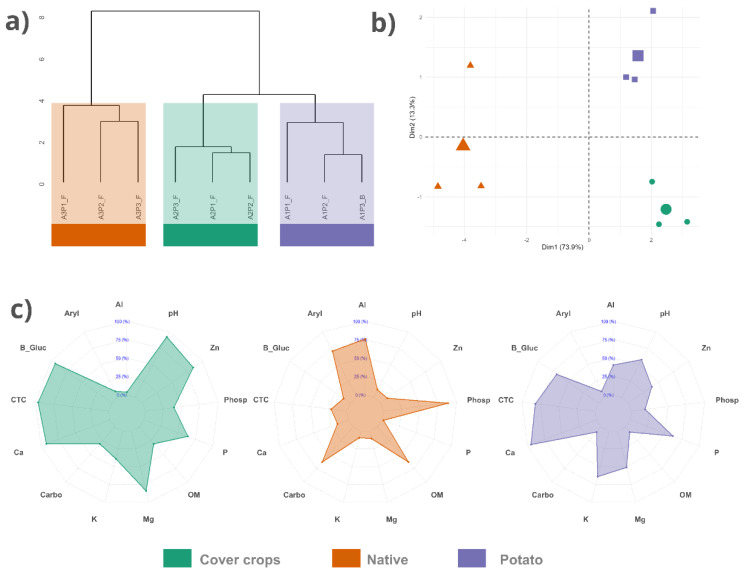
Effect of land use on the chemical and enzymatic properties of soils from different Cerrado areas. (**a**) Cluster analysis dendrogram showing the grouping of samples based on chemical and enzymatic soil properties. (**b**) Principal Component Analysis plot illustrating the separation of samples (points) in the multivariate space. (**c**) Radar chart of 13 chemical and enzymatic soil properties, representing the normalized mean values (0–100%) for each area. Al: aluminum; Ca: calcium; K: potassium; Mg: magnesium; P: phosphorus; Zn: zinc; OM: organic matter; CTC: cation exchange capacity; B_Gluc: B-Glucosidase; Aryl: arylsulfatase; Phosp: phosphatase.

Based on the multivariate analyses and the presented soil profiles (Figure 3a–c; Table A1), the investigated areas exhibited clear differences in chemical and enzymatic soil properties, reflecting the consistent separation of the samples into distinct groups. The dendrogram (Figure 3a) highlights this distinction by clustering samples according to their respective areas. The native area forms a separate clade, whereas the PC and CC areas cluster within the same clade. Complementarily, the PCA (Figure 3b) shows that the first principal axis (Dim1, explaining 73.5% of the variance) strongly separates the NC from the rotational monoculture systems. The radar chart (Figure 3c) summarizes the general patterns observed: the NC displays lower values of pH, Ca, Mg, P, and cation exchange capacity (CEC/CTC), along with higher levels of Al, carbon, organic matter, and enzymatic activity (phosphatase and arylsulfatase), whereas the CC and PC areas exhibit higher pH, Ca, Mg, P, and CEC.

### 3.2. Relative Abundance Across Taxonomic Levels and Functional Lifestyles

**Figure 4:**
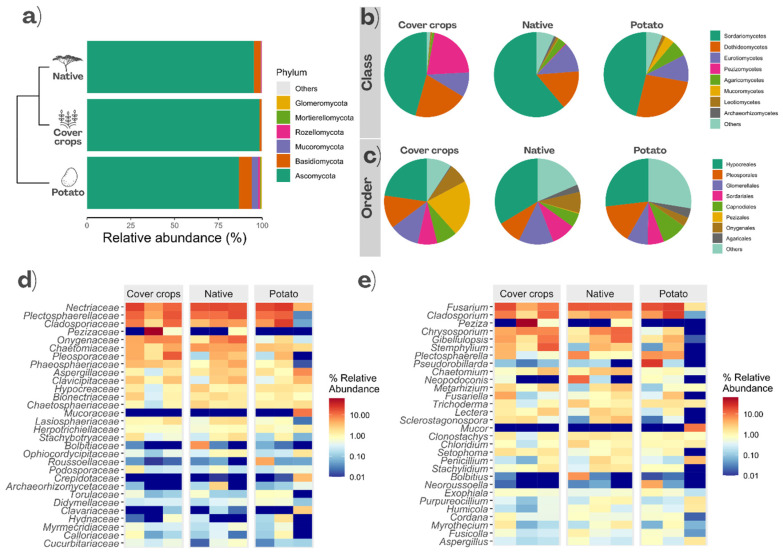
Relative abundances of fungal taxa at different taxonomic levels: phyla (**a**), classes (**b**), orders (**c**), families (**d**), and genera (**e**) in the soil of Cover crops, Native Cerrado, and Potato areas.

Despite the observed differences in soil chemical and enzymatic variables, no significant differences in the relative abundances of fungal taxa among the three areas were detected based on differential abundance analysis using LEfSe. The fungal community was dominated by Ascomycota in all evaluated areas, although its relative abundance varied among environments, being highest in CC (98.4%) and NC (95.2%), and lower in PC (86.7%) (Figure 4a). At the class level, Sordariomycetes (45.7%) and Pezizomycetes (21.1%) predominated in CC, whereas Sordariomycetes (61.1%) prevailed in NC, followed by Dothideomycetes (15.1%) (Figure 4b). In PC, Sordariomycetes (46.2%) remained dominant, accompanied by Dothideomycetes (26%) (Figure 4b). At the order level, Hypocreales was predominant across all areas, reaching the highest proportion in NC (33.7%), followed by PC (26.8%) and CC (22.8%) (Figure 4c). At the family level, Nectriaceae was more abundant in PC (15.3%) and NC (18.4%), but less so in CC (13.4%), where the Pezizaceae predominated (21.1%) (Figure 4d). *Fusarium* was the most abundant genus in NC (14.8%) and PC (13.4%), whereas *Peziza* predominated in CC (21.1%) (Figure 4e).

**Figure 5:**
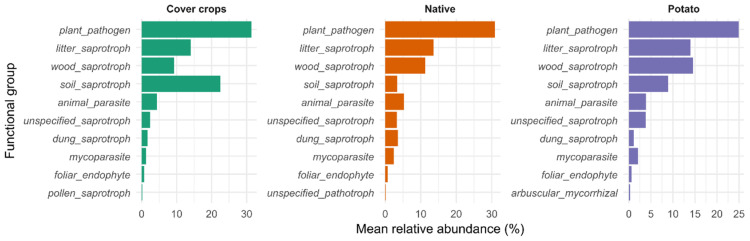
Mean relative abundance (%) of fungal primary lifestyle groups (FungalTraits) across areas.

No significant differences were observed in the relative abundance of fungal functional groups among the studied areas (Kruskal–Wallis test, *p* > 0.05 for all groups) (Figure 5). In CC areas, the most abundant groups were plant_pathogen (31.4%), soil_saprotroph (22.5%), litter_saprotroph (14.1%), wood_saprotroph (9.26%), and animal_parasite (4.38%). In the NC area, the dominant groups were plant_pathogen (30.9%), litter_saprotroph (13.6%), wood_saprotroph (11.3%), animal_parasite (5.29%), and dung_saprotroph (3.57%). In the PC area, the most abundant groups were plant_pathogen (24.9%), wood_saprotroph (14.5%), litter_saprotroph (14.0%), soil_saprotroph (8.93%), and animal_parasite (3.89%).

### 3.3. Alpha and Beta Diversity

**Figure 6:**
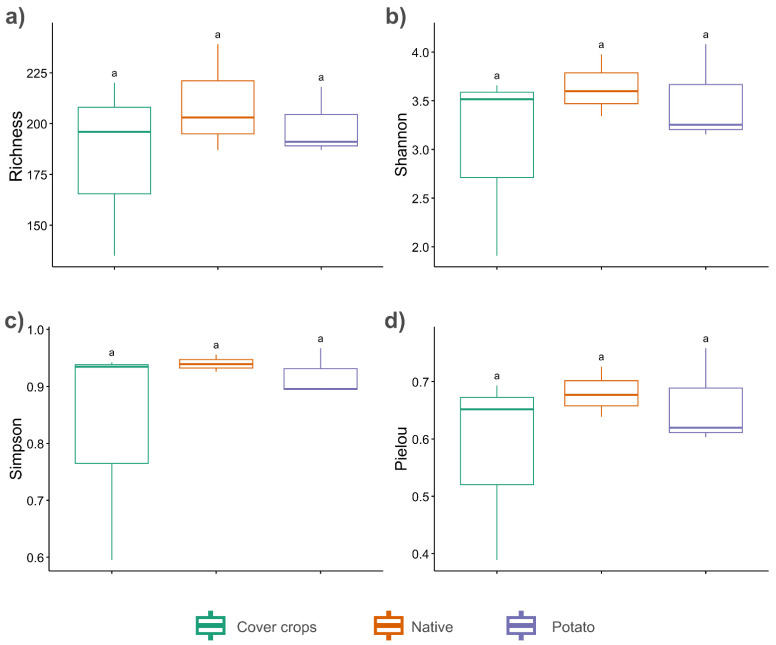
Alpha diversity metrics of soil fungal communities at the genus level in Cover crops, Potato cultivation, and Native Cerrado areas. (**a**) Observed richness; (**b**) Shannon index; (**c**) Simpson index; (**d**) Pielou’s evenness. “a”—there is no statistical difference.

Alpha diversity analyses showed no significant differences among the three evaluated areas (*p* > 0.05) at either the genus or ASV levels. The results at the ASV level are shown in Supplementary Material—Figure S2a–d. At the genus level, the observed richness was slightly higher in the NC area (209.7 ± 26.6), followed by the PC (198.7 ± 16.9) and CC (183.7 ± 43.8) areas (Figure 6a). A similar pattern was found for the Shannon index, with mean values of 3.64 ± 0.32 in the NC area, 3.50 ± 0.51 in the PC area, and 3.03 ± 0.97 in the CC area (Figure 6b). Simpson’s index also indicated higher diversity and lower dominance in the fungal community of the NC area (0.94 ± 0.01), compared to the PC (0.92 ± 0.04) and CC (0.82 ± 0.20) (Figure 6c). Pielou’s evenness followed the same trend, with values of 0.68 ± 0.04 in the NC area, 0.66 ± 0.09 in the PC area, and 0.58 ± 0.16 in the CC area (Figure 6d).

**Figure 7:**
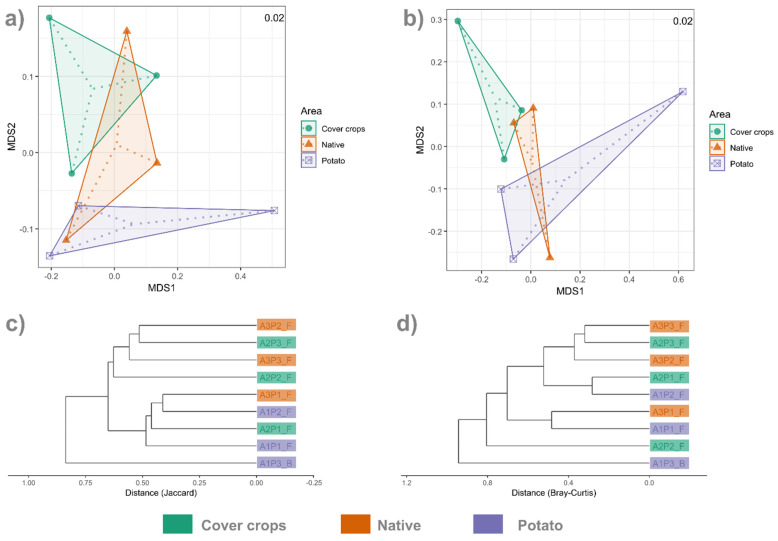
Non-metric Multidimensional Scaling of soil fungal communities at the genus level in Cover crops, Native Cerrado, and Potato areas, based on Bray–Curtis (**a**) and Jaccard (**b**) distance matrices; cluster dendrograms based on Jaccard (**c**) and on Bray–Curtis (**d**) distance matrices.

Beta diversity analyses based on Jaccard and Bray–Curtis dissimilarities revealed no significant differences in fungal community composition among the three evaluated areas (*p* > 0.05). NMDS ordinations for both distance metrics exhibited low stress values (Jaccard = 0.0245; Bray–Curtis = 0.0197), indicating a reliable two-dimensional representation of community dissimilarities (Figure 7a,b). The NMDS plots also show that the native area overlaps with the other areas (Figure 7a,b). Furthermore, NMDS shows a clear separation of the cover crop area and the potato cultivation area. Dendrograms constructed from the two distance matrices did not reveal a clear clustering of samples by area, indicating only subtle variations in community composition among the areas (Figure 7c,d). Multivariate dispersion analysis (β-dispersion) indicated no significant differences in within-group dispersion for both distance metrics (F = 0.29; *p* = 0.692), suggesting homogeneity of variances among areas. Similarly, the ANOSIM test produced low and non-significant R statistics for both Bray–Curtis (R = 0.07; *p* = 0.300) and Jaccard (R = −0.07; *p* = 0.654), supporting the absence of clear compositional separation between environments. Pairwise ANOSIM comparisons between areas also showed non-significant differences (*p* > 0.3). Consistently, PERMANOVA did not detect significant effects of area on fungal community structure for either distance metric (Bray–Curtis: F = 0.94; R^2^ = 0.24; *p* = 0.584; Jaccard: F = 0.94; R^2^ = 0.24; *p* = 0.584).

Analyses were also performed at the ASV level. Multivariate dispersion analysis did not indicate significant differences in within-group dispersion for either distance metric (Bray–Curtis: F = 0.29, *p* = 0.658; Jaccard: F = 0.15, *p* = 0.912), indicating homogeneity among areas. However, NMDS did not present satisfactory stress values (>0.2); therefore, it was replaced by PCoA analysis, which, similarly to the genus level, showed consistent results (Supplementary Material—Figure S3a–d). No significant differences were observed for PERMANOVA (Bray–Curtis: F = 0.94, R^2^ = 0.24, *p* = 0.557; Jaccard: F = 0.77, R^2^ = 0.20, *p* = 0.815) or ANOSIM (Bray–Curtis: R = −0.045, *p* = 0.634; Jaccard: R = −0.045, *p* = 0.589).

### 3.4. Core Mycobiota

**Figure 8:**
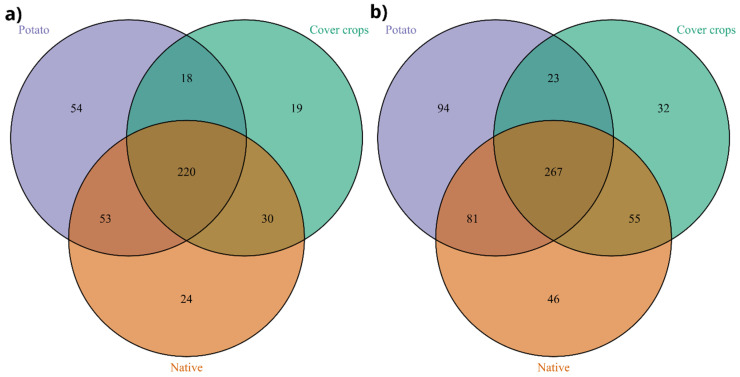
Venn diagrams showing the sharing of fungal taxa among cover crop, native Cerrado, and potato areas at the genus (**a**) and ASV (**b**) levels.

A Venn diagram-based analysis revealed a high level of taxon sharing among fungal communities across the evaluated areas at both taxonomic resolutions (genus and ASV levels) (Figure 8a,b). At the genus level, most taxa were shared among all environments, with 220 genera (96.2%) occurring simultaneously in potato, cover crop, and native areas. In contrast, only a small fraction of genera was exclusive to each environment, comprising 54 (2.1%) in potato, 19 (0.1%) in cover crops, and 24 (0.1%) in native areas. A similar pattern was observed at the ASV level, where 267 ASVs (95.1%) were shared among the three environments. Exclusive ASVs were relatively rare, with 94 (2.4%) in potato, 32 (0.1%) in cover crops, and 46 (0.2%) in native areas.

**Figure 9:**
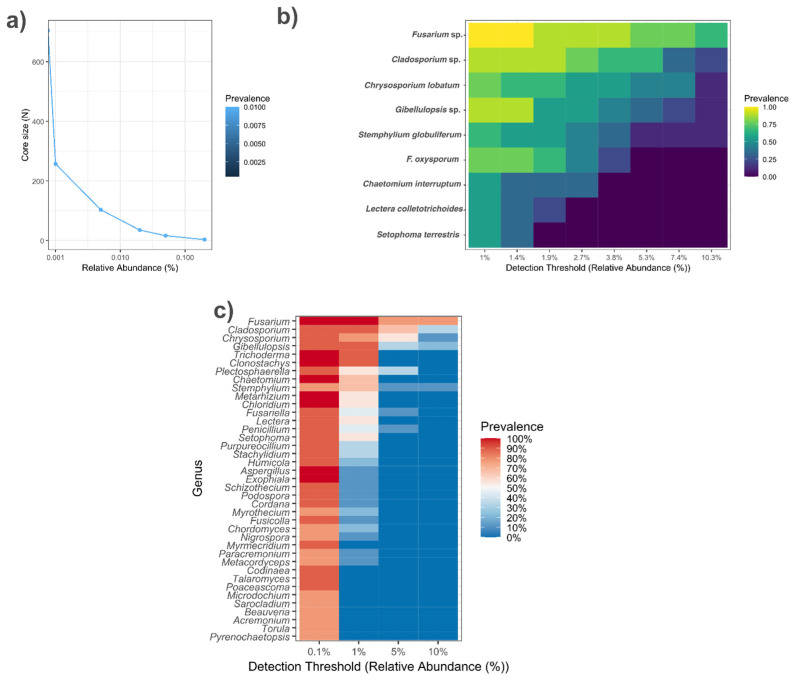
Core soil fungal community structure in Cover crops, Native Cerrado, and Potato cultivation areas. (**a**) Abundance–occurrence curve showing the decrease in core richness with increasing relative abundance thresholds. (**b**) Heatmap of ASVs representing the core mycobiota across detection thresholds (1–2.5% relative abundance). (**c**) Heatmap of genera representing the core mycobiota across detection thresholds.

The core mycobiota was defined using the microbiome package based on prevalence and abundance thresholds, resulting in a set of core taxa that were subsequently aggregated at the genus and/or ASV level (Figure 9). At the ASV level, the core taxa included *Fusarium* sp. (ASV 3), *Cladosporium* sp. (ASV 2), *Chrysosporium lobatum* (ASV 4), and *F. oxysporum* (ASV 10) (Figure 8b). Prevalence analysis showed that *Fusarium* sp. (ASV 3) was present in 100% of the samples, followed by *Gibellulopsis* sp. (ASV 5) and *Cladosporium* sp. (ASV 2) in 80%, and *Chrysosporium lobatum* (ASV 4) and *F. oxysporum* (ASV 10) in 60% of the samples. Based on the established frequency and abundance thresholds, this analysis identified 157 genera as core members, encompassing representatives from multiple orders, including *Fusarium*, *Cladosporium*, *Chrysosporium*, *Trichoderma*, *Clonostachys*, and *Setophoma* (Figure 9b,c).

### 3.5. Correlations Between Fungal Genera Relative Abundance, Primary Lifestyles and Environmental Variables

We conducted correlation analyses between soil chemical and enzymatic variables and both fungal genera and primary lifestyles. No significant associations were detected between environmental variables and fungal lifestyles. In contrast, several fungal genera showed significant positive correlations with soil moisture. *Cladosporium* (ρ = 0.93, adjusted *p* = 0.013), *Sistotrema* (ρ = 0.94, adjusted *p* = 0.008), *Sarocladium* (ρ = 0.91, adjusted *p* = 0.022), *Alternaria* (ρ = 0.96, adjusted *p* = 0.003), *Colletotrichum* (ρ = 0.95, adjusted *p* = 0.008), and *Articulospora* (ρ = 0.94, adjusted *p* = 0.010) were significantly associated with this variable (adjusted *p* < 0.05).

## 4. Discussion

In our study, we evaluated the similarity of soil fungal communities and their chemical and enzymatic characteristics across three environments: a remnant Cerrado area, an adjacent cover crop field, and a potato production area located approximately 18.5 km away. Studies conducted in the Cerrado generally report pronounced differences in microbial communities across native ecosystems and rotational monoculture settings, mainly due to contrasts in organic matter quality, rainfall seasonality, and disturbance history [38,43,44,45]. Surprisingly, the three environments analyzed here exhibited highly similar fungal taxonomic profiles. Although microbial communities in the Cerrado are known to respond sensitively to environmental disturbances such as nutrient enrichment and liming [66,67], as well as to longitudinal and topographic gradients [68,69], most of these studies focus on essentially native areas or regions exposed only to minimal or moderate impacts. In contrast, our study covers a landscape influenced by rotational monoculture systems [48,49], yet no significant differences in the composition or diversity of soil fungal communities were detected, even in the presence of marked variation in soil chemical and enzymatic properties. These results suggest a degree of similarity in soil fungal communities across the studied areas; however, the underlying mechanisms cannot be directly inferred from the present data. Further studies are needed to disentangle the relative contributions of dispersal processes, environmental filtering, and landscape connectivity. These findings also raise questions about a possible process of biotic homogenization [16,18] or edge effects [46,47] influencing soil communities in Cerrado landscapes adjacent to agricultural matrices.

The predominance of Ascomycota, especially the classes Sordariomycetes and Pezizomycetes, across all evaluated areas is consistent with studies reporting the wide distribution and high resilience of these taxa in both natural and managed soils [43,70,71,72,73,74]. In contrast, all other phyla presented relative abundances below 10% in all environments, including Basidiomycota, which has been reported at higher levels in other soil fungal community studies [43,73]. The low representation of Basidiomycota observed here aligns with evidence that this phylum tends to decrease in environments subjected to agricultural land use and soil cover [70,75]. This may occur because basidiomycetes are generally more sensitive to environmental variation [76,77]. In contrast, ascomycetous fungi display greater functional plasticity and a broader metabolic repertoire [76], which enhances their adaptation and persistence in environments undergoing constant change [77].

The composition of the core mycobiota also reinforces this pattern of dominance and resilience among generalist fungi. Genera such as *Fusarium*, *Cladosporium*, *Chrysosporium*, *Trichoderma*, *Clonostachys*, and *Setophoma* were identified as core members, suggesting that certain taxa act as ecological generalists capable of thriving under contrasting environmental conditions and management regimes. Moreover, several of these genera have agronomic relevance. *Fusarium* includes numerous phytopathogenic species that affect crops such as potato [26], whereas *Setophoma* is known to cause root diseases in *Solanum* species [78]. Conversely, *Trichoderma* and *Clonostachys* are widely associated with mycoparasitism and used as biocontrol agents [79]. Species of the genus *Cladosporium* are melanized saprotrophs with broad environmental tolerance and are commonly found in soils and other contrasting habitats [80,81,82]. Overall, the taxa forming the core across the three areas share high dispersal capacity and marked ecological plasticity, traits that promote their persistence in landscapes subject to various forms of disturbance and land use.

We did not find any significant differences in alpha diversity metrics among the three areas. Beta diversity analyses (PERMANOVA and ANOSIM) did not show significant differences among areas, although NMDS and PCoA revealed a separation between the potato and cover crop areas. The dendrograms based on Bray–Curtis and Jaccard distances revealed heterogeneous clustering patterns among samples, with no clear grouping according to environment. The similarity observed between fungal communities from the native Cerrado and adjacent agricultural areas reflects the sharing of many taxa across environments that are, in theory, contrasting. A similar pattern has been reported in other ecosystems, where fungal communities in highly connected habitat fragments were found to be more similar to each other than those in less connected areas [83]. Furthermore, this phenomenon of reduced dissimilarity has been documented in microbial communities from different ecosystems, particularly in landscapes subjected to agriculture or urbanization [15,16,17,18], resulting in a process of biotic homogenization [19,20]. In our study, the flow of propagules across the landscape, among the different areas, likely facilitated the observed increase in similarity among fungal communities. It is already known that fungal spores can be transported from one area to another [84], and that soil microbial communities in closer spatial proximity tend to be more similar [85,86]. However, although this study reveals substantial sharing of fungal taxa between the agricultural areas and the impacted Cerrado remnant, it is important to note that such patterns do not constitute formal evidence of biological homogenization. As discussed by Olden and Rooney [19] and reinforced by more recent reviews [20,87], homogenization can only be quantified when historical data are available to compare species identities before and after disturbance.

The interaction between soil moisture and fungal community diversity is multifaceted, considering that it acts directly as a physiological constrain and indirectly via nutrients, pH or vegetation diversity, which can be considered a nonlinear effect, with optimal diversity at intermediate moisture levels. However, moisture variability might enhance diversity by promoting niche differentiation [33,88,89].

Since our analyses rely exclusively on the current composition of the communities, the results indicate contemporary convergence and reduced spatial taxonomic heterogeneity, but do not allow inferences about temporal changes in the strict sense of biological homogenization. In this context, our data raise an important point for studies and monitoring protocols aimed at continuously assessing Cerrado remnants located adjacent to agricultural areas.

## 5. Conclusions

Overall, our results indicate that native Cerrado remnants embedded within crop rotation matrices may harbor fungal communities that closely resemble those of adjacent managed areas. However, as this study represents a spatially restricted assessment, the observed patterns should be interpreted with caution, and broader temporal and spatial replication will be necessary to capture the full extent of fungal diversity and community dynamics. These findings underscore the need for further research on the mechanisms driving fungal dispersal, edge effects, and the long-term impacts of agricultural land-use on fungal diversity and ecological integrity in the Cerrado.

## Data Availability

The original contributions presented in this study are included in the article. Further inquiries can be directed to the corresponding author.

## Supplementary Materials

The following supporting information can be downloaded at: https://www.mdpi.com/article/10.3390/jof12050346/s1. Table S1: Summary of sequence processing and quality filtering for all samples. Figure S1. Rarefaction curves for all samples and areas (a) Individual rarefaction curves showing species richness (number of ASVs) as a function of sample size (number of reads) for each replicate. (b) Sample-size–based rarefaction and extrapolation curves grouped by land-use area (Cover crops, Native, and Potato). Solid points represent the richness observed at the maximum sampling depth (rarefied values), whereas dashed lines indicate the extrapolated estimates of potential richness for each management system. The stabilization of the curves across all areas indicates high sampling coverage, suggesting that most of the species richness present in the communities was effectively captured. Cover crops: A2P1_F, A2P2_F, A2P3_F; Native: A3P1_F, A3P2_F, A3P3_F; Potato: A1P1_F, A1P2_F, A1P3_B. Figure S2. Alpha diversity metrics of soil fungal communities at the ASV level in Cover crops, Potato cultivation, and Native Cerrado areas. (a) Observed richness; (b) Shannon index; (c) Simpson index; (d) Pielou’s evenness. Figure S3. Principal Coordinate Analysis of soil fungal communities at the ASV level in cover crop areas, native Cerrado, and potato fields, based on Jaccard (a) and Bray–Curtis (b) distance matrices; cluster dendrograms based on Jaccard (c) and Bray–Curtis (d) distance matrices.

## Appendix A

**Table A1:** Soil chemical and enzymatic properties across different areas. Values are presented as mean ± standard deviation. Statistical test results are available at DOI: https://doi.org/10.3390/microorganisms13040804.

Variable	Cover Crops	Native	Potato
Moisture (%)	28.56 ± 1.78	26.25 ± 1.69	34.90 ± 15.95
Ca (cmol_c_ dm^−3^)	5.53 ± 0.35	1.93 ± 0.95	5.67 ± 0.32
Mg (cmol_c_ dm^−3^)	2.53 ± 0.32	0.83 ± 0.32	1.77 ± 0.15
Al (cmol_c_ dm^−3^)	0.97 ± 0.06	2.27 ± 0.38	1.63 ± 0.15
Zn (mg dm^−3^)	30.97 ± 5.50	6.30 ± 3.66	15.47 ± 2.08
pH	6.77 ± 0.23	5.30 ± 0.26	6.13 ± 0.21
Carbon (g kg^−1^)	14.69 ± 0.33	16.82 ± 2.32	13.34 ± 0.58
Aryl (μg PNG g^−1^ soil h^−1^)	104.49 ± 12.70	221.81 ± 48.60	104.82 ± 14.68
B_Gluc (μg PNG g^−1^ soil h^−1^)	145.21 ± 6.36	60.54 ± 9.71	119.56 ± 3.94
Phosp (μg PNG g^−1^ soil h^−1^)	231.76 ± 4.74	250.89 ± 4.11	223.55 ± 9.37
OM (g kg^−1^)	25.33 ± 0.58	29.00 ± 4.00	23.00 ± 1.00
K	201.93 ± 78.72	56.40 ± 38.80	326.30 ± 154.43
CTC	89.82 ± 1.20	58.14 ± 10.30	83.52 ± 0.61
P	275.67 ± 42.83	8.37 ± 8.62	267.67 ± 147.27
