# Supplementary material for: Soil Fungal Communities Across Contrasting Land-Use Systems in an Intensively Managed Cerrado Landscape
Source: J Fungi (Basel). 2026 May 7;12(5):346. doi: 10.3390/jof12050346 (PMC13208492; doi:10.3390/jof12050346)
Supplement: Supplementary file 1 [file jof-12-00346-s001.zip › jof-4202392-supplementary.pdf]

Soil fungal communities across contrasting land-use systems in an intensively managed Cerrado landscape

Jefferson Brendon Almeida dos Reis; Thayssa Monize Rosa de Oliveira; Samia Gomes-da-Silva; Maria Regina Sartori; Fabyano Alvares Cardoso Lopes; Alessandra Monteiro de Paula; Nadson de Carvalho Pontes; Helson Mario Martins do Vale

Table S1. Summary of sequence processing and quality filtering for all samples

sample-id	Area	Raw	After bbduk	Filtered	Input passed filter (%)	Denoised	Merged	Input merged (%)	Non-chimeric	Input non-chimeric (%)
A1P1_F	Potato	157310	148182	148182	100	145183	133982	90.42	127081	85.76
A1P2_F	Potato	156422	148242	148242	100	146455	134655	90.83	125770	84.84
A1P3_B	Potato	161702	153727	153727	100	151696	133088	86.57	127129	82.7
A2P1_F	Cover crops	164834	156181	156181	100	154401	141675	90.71	132879	85.08
A2P2_F	Cover crops	187026	176681	176681	100	175949	171421	97.02	161328	91.31
A2P3_F	Cover crops	143228	135174	135174	100	133915	126152	93.33	113393	83.89
A3P1_F	Native	135832	128751	128751	100	127455	119861	93.1	112059	87.04
A3P2_F	Native	102239	96254	96254	100	94250	86567	89.94	83719	86.98
A3P3_F	Native	168948	159770	159770	100	157669	141472	88.55	116064	72.64

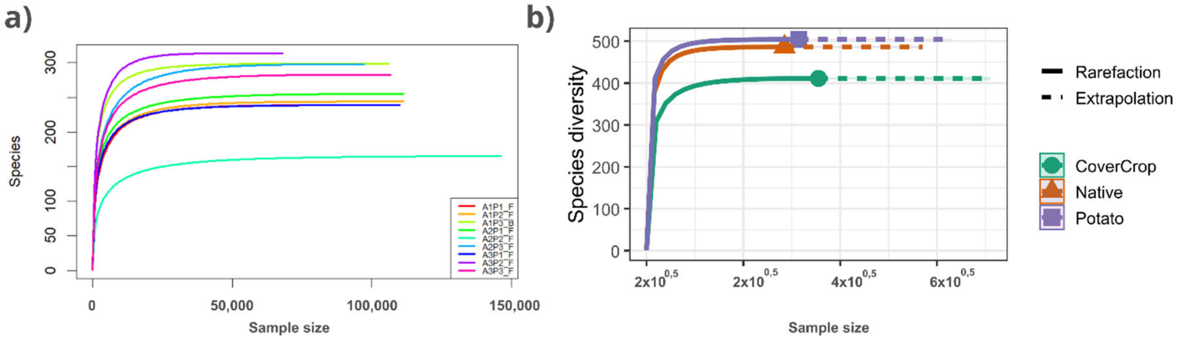

Figure S1. Rarefaction curves for all samples and areas a) Individual rarefaction curves showing species richness (number of ASVs) as a function of sample size (number of reads) for each replicate. b) Sample-size–based rarefaction and extrapolation curves grouped by land-use area (Cover crops, Native, and Potato). Solid points represent the richness observed at the maximum sampling depth (rarefied values), whereas dashed lines indicate the extrapolated estimates of potential richness for each management system. The stabilization of the curves across all areas indicates high sampling coverage, suggesting that most of the species richness present in the communities was effectively captured. Cover crops: A2P1_F, A2P2_F, A2P3_F; Native: A3P1_F, A3P2_F, A3P3_F; Potato: A1P1_F, A1P2_F, A1P3_B.

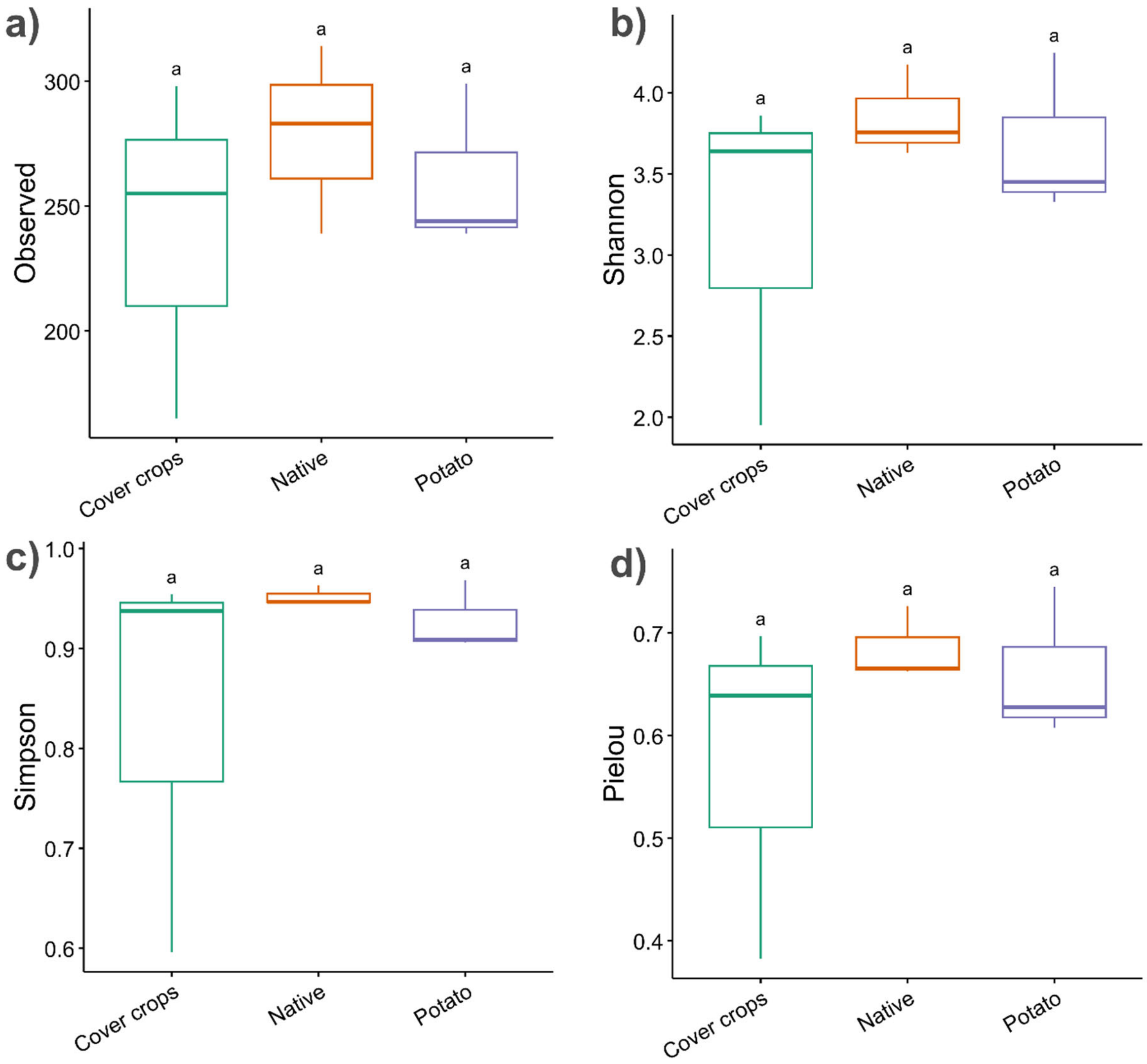

Figure S2. Alpha diversity metrics of soil fungal communities at the ASV level in Cover crops, Potato cultivation, and Native Cerrado areas. a) Observed richness; b) Shannon index; c) Simpson index; d) Pielou's evenness.

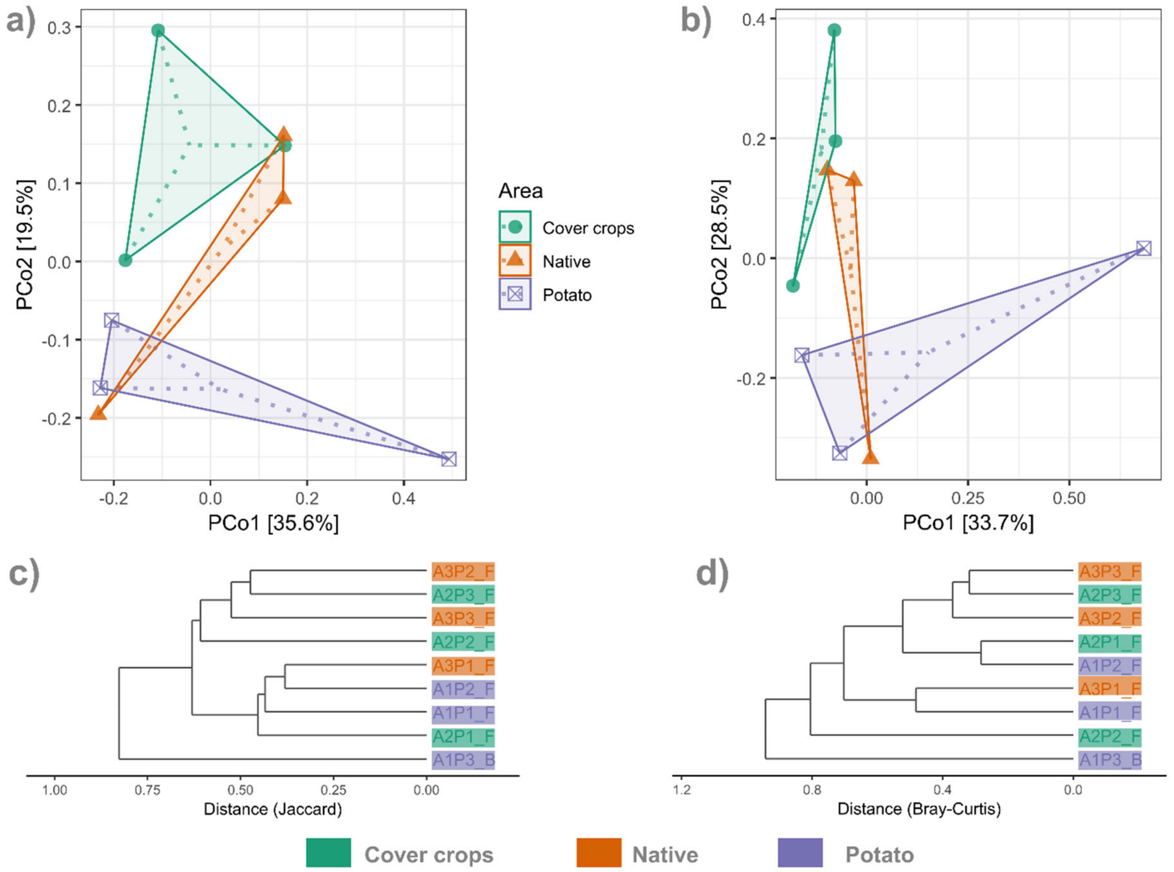

Figure S3. Principal Coordinate Analysis of soil fungal communities at the ASV level in cover crop areas, native Cerrado, and potato fields, based on Jaccard (a) and Bray–Curtis (b) distance matrices; cluster dendrograms based on Jaccard (c) and Bray–Curtis (d) distance matrices
